# Curcumin Attenuates Beta-Amyloid-Induced Neuroinflammation via Activation of Peroxisome Proliferator-Activated Receptor-Gamma Function in a Rat Model of Alzheimer's Disease

**DOI:** 10.3389/fphar.2016.00261

**Published:** 2016-08-19

**Authors:** Zun-Jing Liu, Zhong-Hao Li, Lei Liu, Wen-Xiong Tang, Yu Wang, Ming-Rui Dong, Cheng Xiao

**Affiliations:** ^1^Department of Neurology, China-Japan Friendship HospitalBeijing, China; ^2^Laboratory of Immunology and Equipment, Institute of Clinical Medicine Science, China-Japan Friendship HospitalBeijing, China

**Keywords:** Alzheimer's disease, curcumin, neuroinflammation, peroxisome proliferator-activated receptor-gamma, NF-κB

## Abstract

Neuroinflammation is known to have a pivotal role in the pathogenesis of Alzheimer's disease (AD), and curcumin has been reported to have therapeutical effects on AD because of its anti-inflammatory effects. Curcumin is not only a potent PPARγ agonist, but also has neuroprotective effects on cerebral ischemic injury. However, whether PPARγ activated by curcumin is responsible for the anti-neuroinflammation and neuroprotection on AD remains unclear, and needs to be further investigated. Here, using both APP/PS1 transgenic mice and beta-amyloid-induced neuroinflammation in mixed neuronal/glial cultures, we showed that curcumin significantly alleviated spatial memory deficits in APP/PS1 mice and promoted cholinergic neuronal function *in vivo* and *in vitro*. Curcumin also reduced the activation of microglia and astrocytes, as well as cytokine production and inhibited nuclear factor kappa B (NF-κB) signaling pathway, suggesting the beneficial effects of curcumin on AD are attributable to the suppression of neuroinflammation. Attenuation of these beneficial effects occurred when co-administrated with PPARγ antagonist GW9662 or silence of PPARγ gene expression, indicating that PPARγ might be involved in anti-inflammatory effects. Circular dichroism and co-immunoprecipitation analysis showed that curcumin directly bound to PPARγ and increased the transcriptional activity and protein levels of PPARγ. Taking together, these data suggested that PPARγ might be a potential target of curcumin, acting to alleviate neuroinflammation and improve neuronal function in AD.

## Introduction

Alzheimer's disease (AD) is a chronic neurodegenerative disease and the most common form of dementia. It is characterized by selective neuronal loss, progressive memory, and cognitive impairment (Campbell and Gowran, 2007). The molecular pathogenesis of AD involves extracellular deposition of beta-amyloid (Aβ) peptides in the hippocampus. Aggregated Aβ can trigger microglia and astrocytes activation, leading to the production of inflammatory factor, including nitric oxide (NO), tumor necrosis factor-α (TNF-α), interleukins (ILs), and prostaglandins, in the vicinity of Aβ peptides, which may cause neuronal death (Akiyama et al., 2000; Kitazawa et al., 2004). This inflammatory response increases as the disease progresses, which eventually leads to neurodegeneration. Moreover, epidemiological study suggested that inflammation is a key player in the pathophysiology of AD (Szekely et al., 2004). Consistently, non-steroidal anti-inflammatory drugs (NSAIDs) suppress neuroinflammatory response in a dose-dependent manner and reduce behavioral deficits in transgenic animal models of AD (McGeer and McGeer, 2007). These studies demonstrate the role of inflammation in the pathogenesis of AD and provide the rational of anti-inflammatory therapy.

Many studies have been conducted to determine the potential therapeutics to ameliorate AD. Increasing studies have shown that curcumin has therapeutic effects for AD (Cole et al., 2007). Curcumin is known to reduce Aβ oligomer and fibril formation (Yang et al., 2005; Xiong et al., 2011), inhibit the neurotoxicity of Aβ in the brain (Jiang et al., 2012; Sun et al., 2014), suppress Aβ-induced inflammation (Lim et al., 2001; Lu et al., 2014) and markedly reduce the levels of IL-1β (Griffin et al., 1998) and iNOS (Begum et al., 2008) in transgenic mouse brain. Clinical trial demonstrated that curcumin has beneficial on AD patients (Baum et al., 2008). Despite the promising prospects, the exact mechanism which curcumin exerts its neuroprotection largely remains unknown.

Furthermore, a mechanistic study showed that anti-inflammatory effects can be achieved by inhibiting the nuclear factor kappa B (NF-κB) (Becaria et al., 2003) and ERK (Giri et al., 2004) signaling pathways, which can be regulated by peroxisome proliferator-activated receptor gamma (PPARγ). The actions of PPARγ and its agonists in AD have been well documented over the past decade. Treatment with PPARγ agonist lead to the reduced Aβ production, neuroinflammation, and improvement of cognitive function (Sodhi et al., 2011; Mandrekar-Colucci et al., 2012). One proposed mechanism for the actions of PPARγ is that the anti-inflammatory effects of PPARγ linked to cognitive impairment. Our previous study demonstrated that curcumin is a potent PPARγ agonist (Liu et al., 2011), and has neuroprotective effects on ischemic injury *in vitro* and *in vivo* (Liu et al., 2013, 2014). However, whether the activation of PPARγ of curcumin is responsible for its neuroprotection on AD remains unclear and needs to be further investigated.

## Materials and methods

### Chemicals and reagents

Curcumin, GW9662, Aβ_1–42_, and Griess reagent were purchased from Sigma. Dulbecco's Modified Eagle Medium Nutrient Mixture F-12 (DMEM/F-12), fetal bovine serum (FBS), and Opti- Minimum Essential Medium (MEM) were producted by Gibco. PPARγ siRNA was synthesized by Invitrogen. Lipofectamine LTX and Plus Reagent was produced by Invitrogen. Choline acetyltransferase (ChAT), glial fibrillary acidic protein (GFAP), Iba-1, NF-κB p65, IκBα, and PPARγ antibodies were obtained from Abcam. IL-1β, TNF-α, and COX-2 ELISA kits were purchased from R&D Company. A choline/acetylcholine (Ach) assay kit was supplied by Abcam. A ChAT ELISA kit was obtained from MyBioSource Inc. A PPARγ transcription factor assay kit and PPARγ ligand binding domain (human recombinant) were purchased from Cayman Chemical. A co-immunoprecipitation (Co-IP) kit produced by Pierce was used, and LDH assay kit was supplied by Nanjing Jiancheng Bioengineering Institute.

### Animals and treatment

Transgenic mice overexpress Swedish mutant AβPP695 and deletion of exon-9 mutant PS1 (APPswe/PS1Δ9). Mice were housed in a 12 h light/dark room at 24°C in the Animal Center of Chinese Academy of Medical Sciences. 150 mg/kg of curcumin and 4 mg/kg of PPARγ inhibitor GW9662 (Garrido-Gil et al., 2012) were dissolved in 10% dimethyl sulfoxide (DMSO), and intraperitoneally injected to APP/PS1 double-transgenic 8-month-old mice daily for 4 consecutive weeks. The animal experiments were approved by the animal experimental ethics committee of China-Japan Friendship Hospital.

### Hippocampal neuronal/glial culture and treatment

Primary hippocampus neuronal/glial cultures were obtained from the brain of rat embryos at 19 d of gestation (Beijing Vital River Laboratory Animal Technology Co. Ltd., Beijing, China). The procedure was approved by the Animal Ethic Committee of China-Japan Friendship Hospital. In brief, the hippocampus was isolated, and then incubated with 0.25 mg/mL trypsin at 37°C for 30 min, gently triturated in DMEM/F-12, and centrifuged to collect the cells. Dissociated cells were plated in dishes coated with 10 mg/mL poly-D lysine and grown in DMEM/F-12 with 10% FBS, 1 mM sodium pyruvate, 0.1 mM non-essential amino acids, 2 mM L-glutamic acid, 100 U/mL penicillin, and 100 μg/mL streptomycin in incubator with 95% air/5% CO_2_ at 37°C. Cultures grown for 7 d *in vitro* were used for experiments. Aβ_1–42_ solution (dissolved in PBS) was placed at 37°C with gentle shaking for 72 h to allow the peptide to aggregate. Cells were pre-treated with 10 μM curcumin, and 25 μM Aβ_1–42_ was added to the media 1 h later, the cells were harvested 24 h later. To inhibit PPARγ function, 1 μM GW9662 (dissolved in DMSO) was incubated with the cultures or PPARγ siRNA was transfected to cells 1 h prior to Aβ_1–42_ treatment, and the cells were harvested 24 h later.

### Silencing of PPARγ by RNAi

Mixed neuronal/glial cultures were grown in a flask to ~60% confluence, and transfected with an optimized concentration of PPARγ siRNA. Transfection was performed using the Lipofectamine LTX and Plus Reagent and 25 nM appropriate PPARγ siRNA, according to the manufacturer's instructions. Transfection was conducted 1 h before Aβ_1–42_ treatment. Whole cell lysates were then prepared, and PPARγ knockdown was confirmed by western blot analysis.

### Morris water maze test

Spatial learning and memory of mice were assessed by the Morris water maze (Institute of Materia Medica, Chinese Academy of Medical Sciences and Peking Union Medical College, Beijing, China) after the mice had received curcumin for continuous 8 weeks. The Morris water maze test was performed in a round pool (diameter 120 cm and depth 40 cm) filled with nontoxic opaque water (Hernandez-Perez et al., 2015; Singh and Kumar, 2015). The water maze was divided into four quadrants. A platform with diameter of 10 cm was placed in the pool. The water was filled in the pool until the platform was 2 cm below the water surface. The water temperature was maintained at about 23°C. The protocol was fixed and maintained throughout four acquisition trials, except for randomly selecting starting point. At the start of each trial, each mouse was allowed to swim in the water at one of the four quadrants for a maximum of 120 s to find the platform. After finding the platform, the mouse was kept on the platform for 30 s, and would be placed on the platform for 30 s. Each mouse received four trials every day. The latency to find the platform (escape latency), the swimming distance, and swimming speed were recorded. The training period was conducted for 5 consecutive days in which the platform was kept as the same. The latency to escape was calculated as the average time to find the platform of the four trials within 1 d.

Memory retention was evaluated on day 6 with a probe trial in which the platform was removed. The mice were placed in the pool and allowed to swim freely for 120 s and the crossing number of the platform and time of mouse in the destination were recorded.

### Immunohistochemical analysis

Mice were anesthetized with hydrate chloral and perfused via the ascending aorta first with 0.9% saline and then followed by 0.1 M PBS (pH 7.4), which contains 4% paraformaldehyde. Then the whole brains were removed and fixed in the 0.1 M PBS (pH 7.4) containing 4% paraformaldehyde and 30% sucrose for 4 h. The brains were cut into sections of 40-μm. Six serial sections were taken and incubated with the ChAT, GFAP, or Iba-1 antibodies, respectively. And then the sections were incubated with the biotinylated secondary antibodies for 90 min. The immunoreactivity was visualized by 0.01% hydrogen peroxidase and 0.03% 3, 3′-diaminobenzidine (DAB). The light microscopy (NIKON E600, Japan) were used to observe sections, and the intensity of the stained area of each group was analyzed using an Image-Pro plus system (Media Cybernetics, Silver Spring, MD, USA). All evaluations were performed by a researcher blind to the experimental design.

### ELISA assay of inflammatory mediators

Immediately after mice were decapitated, hippocampi were isolated, dissected, homogenized and centrifuged, the supernatant was collected. For cytokine assay in the cell media, the culture supernatants were collected. IL-1β, TNF-α, and COX-2 were measured by ELISA kits according to the manufacturer's instructions (Spatuzza et al., 2014).

### Western blot assay

Mouse hippocampus and cells were lysed on ice for 15 min in lysis buffer, which then were centrifuged at 12,000 g at 4°C for 15 min to collect the supernatants. Protein concentration was measured with Bradford protein assay. Samples containing 50 μg proteins were loaded with loading buffer and separated by 10% sodium dodecyl sulfate-polyacrylamide gel electrophoresis (SDS-PAGE). The separated protein transferred to PVDF membranes and blocked in 5% skim milk-TBST (20 mM Tris-HCl, 500 mM NaCl, 0.1% Tween 20) for 1 h. GFAP, Mac-1, NF-κB p65, IκBα, and PPARγ primary antibodies (dilution again) were added in 5% skim milk-TBST, and incubated overnight at 4°C. The membranes were incubated with secondary antibody in TBST for 2 h at room temperature. The immunoblot was detected with a LAS3000 chemiluminescence system (Fujifilm, Tokyo, Japan), and the densities of the bolt bands were quantified with Gel-Pro Analyzer 4.0 software.

### Ach and ChAT assay

The Ach levels were measured by a choline/Ach assay kit according to the manufacturer's instructions. In brief, the hippocampus was lysed in choline assay buffer by homogenization on ice. Choline assay buffer (46 μL), choline probe (2 μL), and choline enzyme mix (2 μL) were combined to prepare a reaction mixture. Approximately 50 μL of sample was added and incubated for 30 without exposure to light. The absorbance was measured at the wavelength 570 nm.

ChAT assay was performed using a ChAT ELISA kit following the manufacturer's instructions. In brief, the hippocampi or cells were lysed in PBS with an ultrasonic cell disrupter to prepare the samples. Lysates (100 μL) were added to each well. Approximately 100 μL of Detection Reagent A or Detection Reagent B was added to the wells, which were then incubated. After adding 50 μL of Stop Solution, the plates were immediately read at 450 nm.

### Cell immunocytochemistry and immunofluorescence assay

Cells were fixed with 4% paraformaldehyde on cover ships at room temperature for 15 min and washed with PBS for three times. The cells were permeabilized with PBS containing 0.1% Triton X-100 for 10 min, and blocked in 3% normal goat serum for 2 h. The cells were incubated overnight with GFAP (1:500) or Iba-1 (1:500). Cells were subsequently incubated with FITC-conjugated affinipure secondary antibody (1:250). Fluorescent intensity was imaged with an Olympus FV1000 (Olympus, Tokyo, Japan).

### PPARγ transcriptional activity assay

PPARγ transcriptional activity was tested by a PPARγ transcription factor assay kit, which is a sensitive method for detecting specific DNA binding transcription factor activity in nuclear extracts. A specific double-stranded DNA sequence containing PPRE was immobilized onto the bottom of the well of a 96-well plate. PPARγ transcriptional activity assay was performed according to the manufacturer's protocol. In brief, 90 μL of complete transcription factor binding assay buffer was added to the plate, followed by 10 μL of nuclear extracts, which were prepared using a nuclear-cytosol extraction kit. One hundred microliters of diluted PPARγ primary antibody (1:100) was added, and incubated for 1 h at room temperature. HRP conjugate secondary antibody was added and incubated for 1 h at room temperature. One hundred microliters of transcription factor developing solution was added to the samples, and incubated for 30 min with gentle agitation without light. After adding the stop solution, the absorbance was read at 450 nm.

### Measurement of LDH releasing

The culture media were collected, and neuronal injury was assessed by measurement of LDH releasing using LDH kit. The optical density was read at 492 nm. Data were expressed as percentage of optical density of control cells.

### Nitrite assay

NO production was determined by measuring the amount of nitrite (${\text{NO}}_{2}^{-}$) accumulated in supernatants of mixed neuronal/glial cultures, which was detected by Griess assay as described previously.

### Co-IP assay

Nuclear extracts of primary cultured cells were prepared using nuclear-cytosol or a membrane extraction kit. The Co-IP assay was conducted according to the protocol of Co-IP kit. Purified PPARγ (300 μg) antibody was immobilized in 100 μL antibody coupling gel. Samples (300 μg proteins) were incubated with gentle shaking for 2 h. The immunoprecipitated complexes were eluted three times with elution buffer, and then subjected to SDS-PAGE. The blot was transferred to a PVDF membrane, incubated with NF-κB or PPARγ antibody, respectively, and detected by an enhanced LAS3000 chemiluminescence system.

### Circular dichroism (CD)

PPARγ protein were dissolved in phosphate buffer (pH 7.40, 0.01 M, *I* = 0.1) to the concentration of 6 μM. Curcumin was dissolved in 1.2 μM solution with methanol with gently shaken. The stock solutions of PPARγ and curcumin were mixed at the ratio of 1:5 (v:v), and detected 5 min later. CD spectra were measured on Jasco-J815 CD spectrometer equipped with a Jasco PTC-423S/15 temperature controller between 260 and 200 nm using a 10 mm cuvette at 37°C.

### Statistical analysis

Statistical analysis was performed with SPSS version 21.0. All data were presented as the mean ± standard deviation (SD). Statistical analysis was carried out on three or more groups using one-way analysis of variance (ANOVA) and multiple comparison tests. Values of *P* < 0.05 were considered statistically significant.

## Results

### Curcumin alleviated spatial learning and memory deficits in APP/PS1 mice

**Figure 1:**
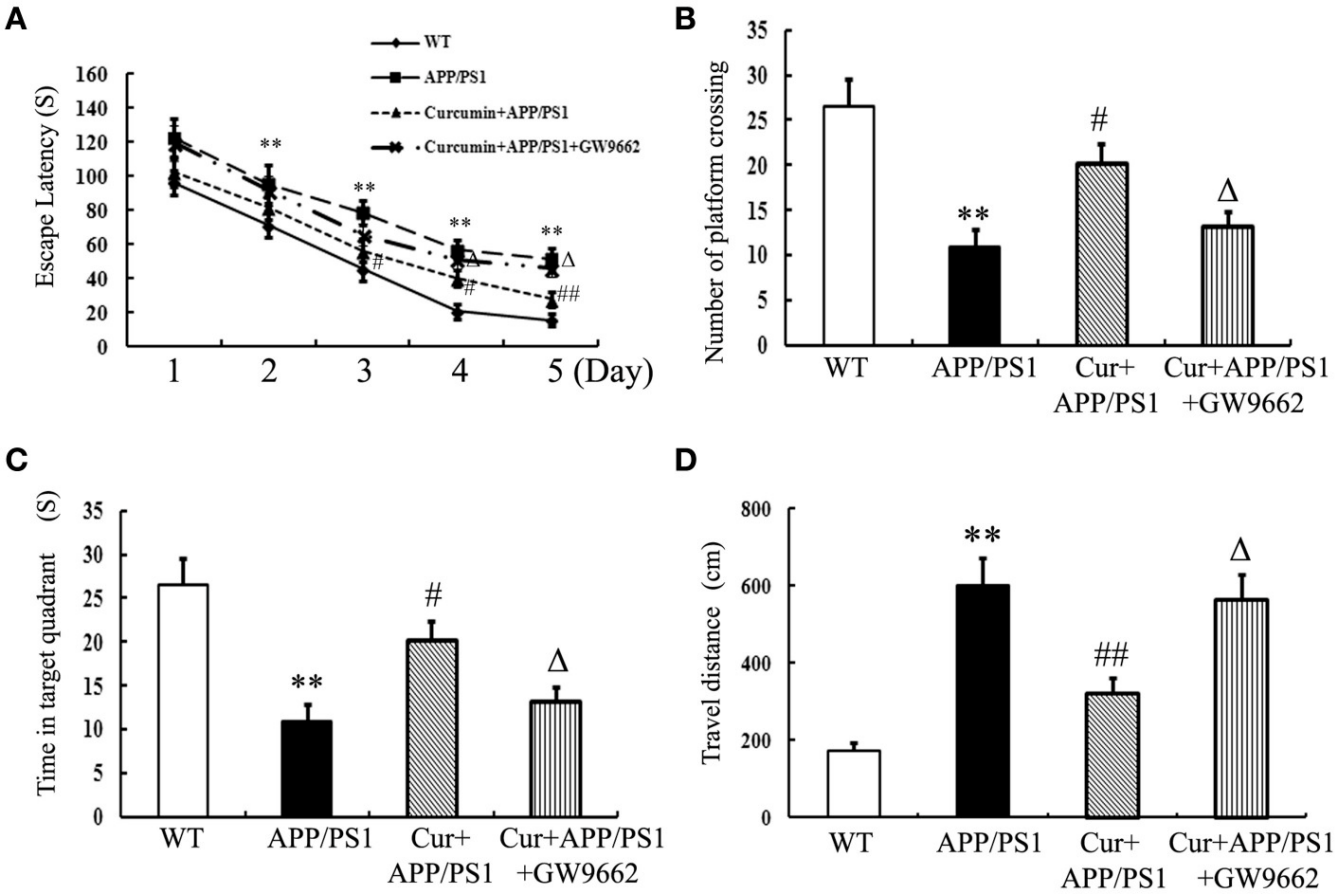
**Curcumin alleviates spatial learning and memory deficits in APP/PS1 mice**. Curcumin 150 mg/kg and PPARγ inhibitor GW9662 4 mg/kg were i.p. injected to APP/PS1 double-transgenic mice for 4 consecutive weeks, and the learning and memory ability was accessed by Morris water maze test. **(A)** The latencies of mice to find the destination. **(B)** Number of platform crossing in probe test. **(C)** Time in the target quadrant in probe test. **(D)** Travel distance in probe test. Results were expressed as mean ± SD. ^**^P < 0.01 vs. WT mice, ^#^*P* < 0.05, ^##^*P* < 0.01 vs. APP/PS1 transgenic mice, ^Δ^*P* < 0.05 vs. curcumin treated mice. *n* = 10 in each group.

Memory deficits were started to show in the 8-month-old APP/PS1 transgenic mice as indicated by longer escape latencies in Morris water maze test (Figure S1) and Aβ accumulation in the hippocampi (Figure S2) was also observed, suggesting that the APP/PS1 transgene caused memory deficits in mice. Curcumin treatment was initiated when APP/PS1 double-transgenic mice were 8 months old. Curcumin markedly decreased the escape latency from day 3 to 5 in the training experiment (Figure 1A). In the probe test, the memory of APP/PS1 mice significantly decreased, such as decreased number of platform crossing, time spent in the target quadrant, and increased travel distances. Curcumin remarkably increased platform crossing number and time spent in the target quadrant, and decreased the travel distance (Figures 1B–D). The improvement in memory function was attenuated by co-administration of GW9662, an inhibitor of PPARγ. Our pilot study showed that intraperitoneally injection of GW9662 (4 mg/kg, dissolved in 10% DMSO) alone daily for 5 weeks did not influence memory (Figure S3) and neuronal function of AD mice (Figure S4). These data suggest that curcumin exert neuroprotective effects on memory deficits of APP/PS1 transgenic mice, and that the neuroprotection of curcumin on AD is closely related to PPARγ.

### Curcumin protected cholinergic neurons in APP/PS1 mice

**Figure 2:**
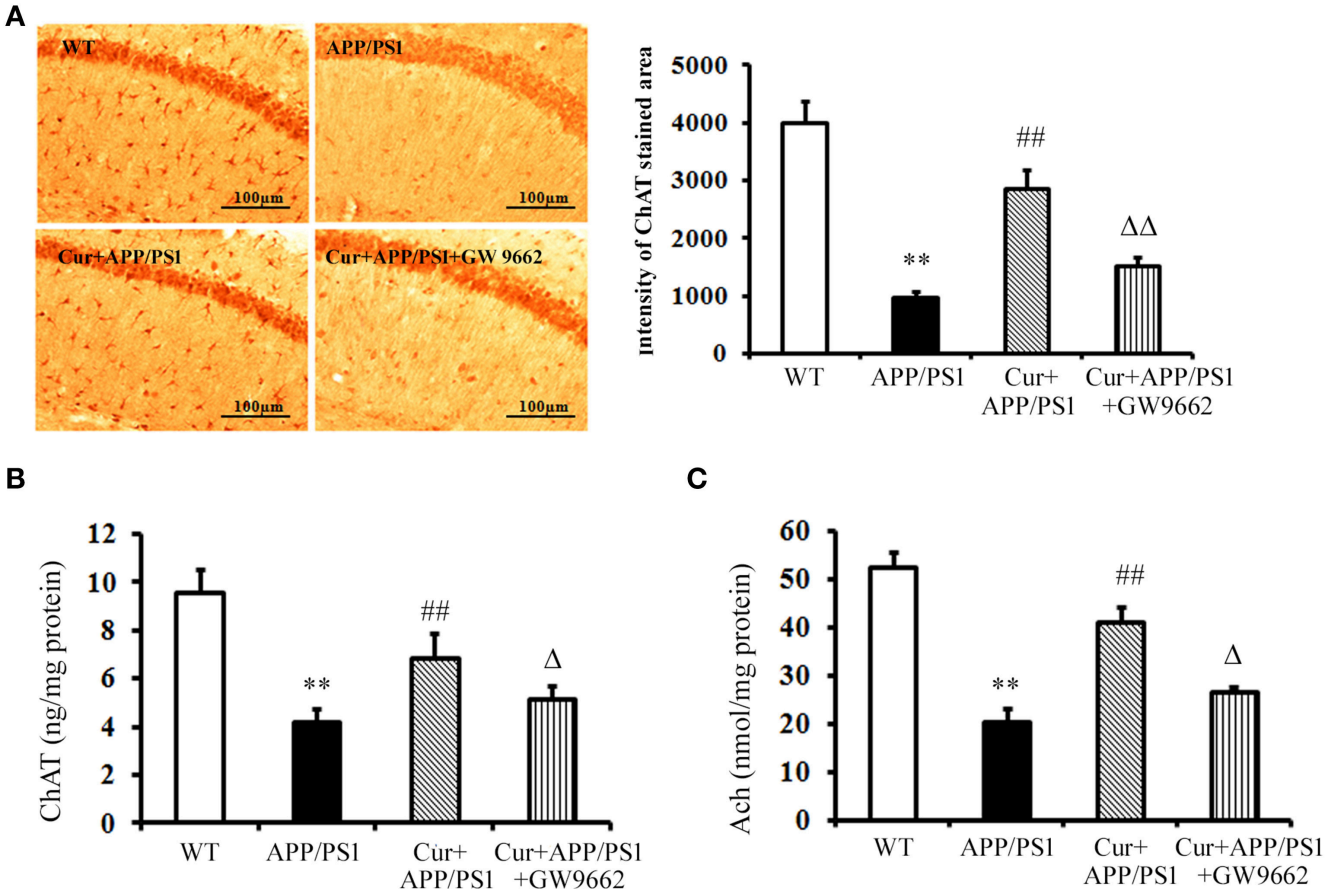
**Curcumin protected cholinergic neurons in hippocampus of APP/PS1 double transgenic mice**. Curcumin 150 mg/kg and PPARγ inhibitor GW9662 4 mg/kg were i.p. injected to APP/PS1 double-transgenic mice for 4 consecutive weeks. **(A)** Immunohistochemistry of ChAT in hippocampus. Representative sections of hippocampus from five mice were shown. **(B)** ELISA assay of ChAT. The results were obtained from six independent experiments. **(C)** Colorimetric analysis of Ach. The results were obtained from six independent experiments. Results were expressed as mean ± SD. ^**^P < 0.01 vs. WT mice, ^##^*P* < 0.01 vs. APP/PS1 transgenic mice, ^Δ^*P* < 0.05, ^ΔΔ^*P* < 0.01 vs. curcumin treated mice.

Cholinergic neurons play a key role in memory function, and the progressive disruption of cholinergic function underlies much of the short-term memory loss observed in AD. The activity of the ChAT transferase enzyme responsible for the synthesis of Ach also decreased in AD. Immunohistochemical and ELISA assays showed that both ChAT-positive cells and ChAT levels declined in the hippocampi of APP/PS1 mice, and increased ChAT-positive cells and ChAT levels were observed upon curcumin treatment (Figures 2A,B). ChAT dysfunction led to the reductions in Ach in the hippocampus, which was reversed and elevated by curcumin treatment (Figure 2C). Co-administration of GW9662 attenuated the beneficial effects of curcumin on cholinergic neurons in the hippocampi of APP/PS1 mice (Figure 2). These data further indicate that PPARγ was involved in the neuroprotection of curcumin on cholinergic neurons *in vivo*.

### Curcumin protected cholinergic neurons in mixed neuronal/glial cultures

**Figure 3:**
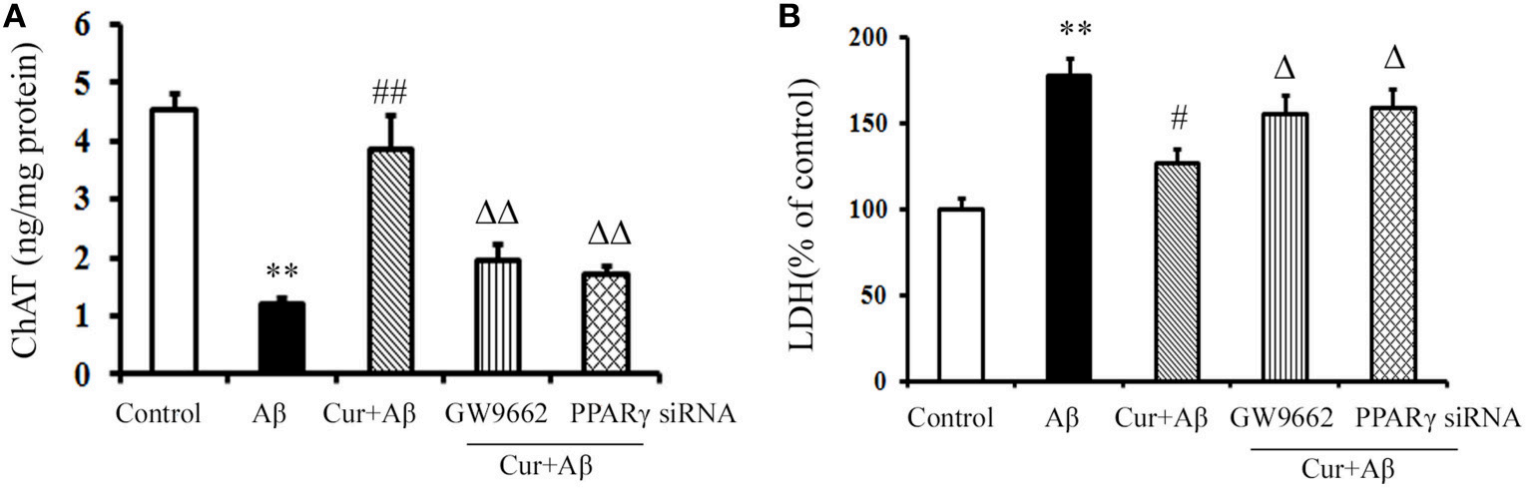
**Curcumin protected cholinergic neurons in mixed neuron/glia cultures**. Mixed neuron/glia cultures were pre-treated with curcumin 10 μM, 1 h later, Aβ_1–42_ 25 μM was added to the mixed cultures. GW9662 1 μM was added into the cultures or cells were transfected with PPARγ siRNA 1 h before Aβ_1–42_ treatment. **(A)** ELISA assay of ChAT. The results were obtained from six independent experiments. **(B)** LDH releasing to the culture medium. Data were expressed as mean ± SD with six individual experiments. ^**^P < 0.01 vs. control cells, ^#^*P* < 0.05, ^##^*P* < 0.01 vs. Aβ_1–42_-challenged cells, ^Δ^*P* < 0.05, ^ΔΔ^*P* < 0.01 vs. curcumin treated cells.

We further investigated the neuroprotective effects of curcumin treatment on cholinergic neurons *in vitro*. As shown in Figure 3, ChAT levels were markedly reduced in Aβ_1–42_-challenged mixed neuronal/glial cultures. Pre-treatment of curcumin increased ChAT levels. LDH is an important marker of neuronal injury. In the present study, Aβ_1–42_ caused neuronal death by activating the inflammatory response, as indicated by elevated LDH levels in the media, suggesting increased cell destruction. However, neuronal death was alleviated by curcumin, which resulted in decreased LDH. The neuroprotection of curcumin was reversed by the treatment of GW9662 or silence of PPARγ. Treatment of cells with GW9662 or PPARγ siRNA alone did not affect cholinergic neuronal function (Figure S5). These results suggested PPARγ is involved in the beneficial effects of curcumin on cholinergic neurons *in vitro*.

### Curcumin suppressed the neuroinflammatory response in APP/PS1 mice

**Figure 4:**
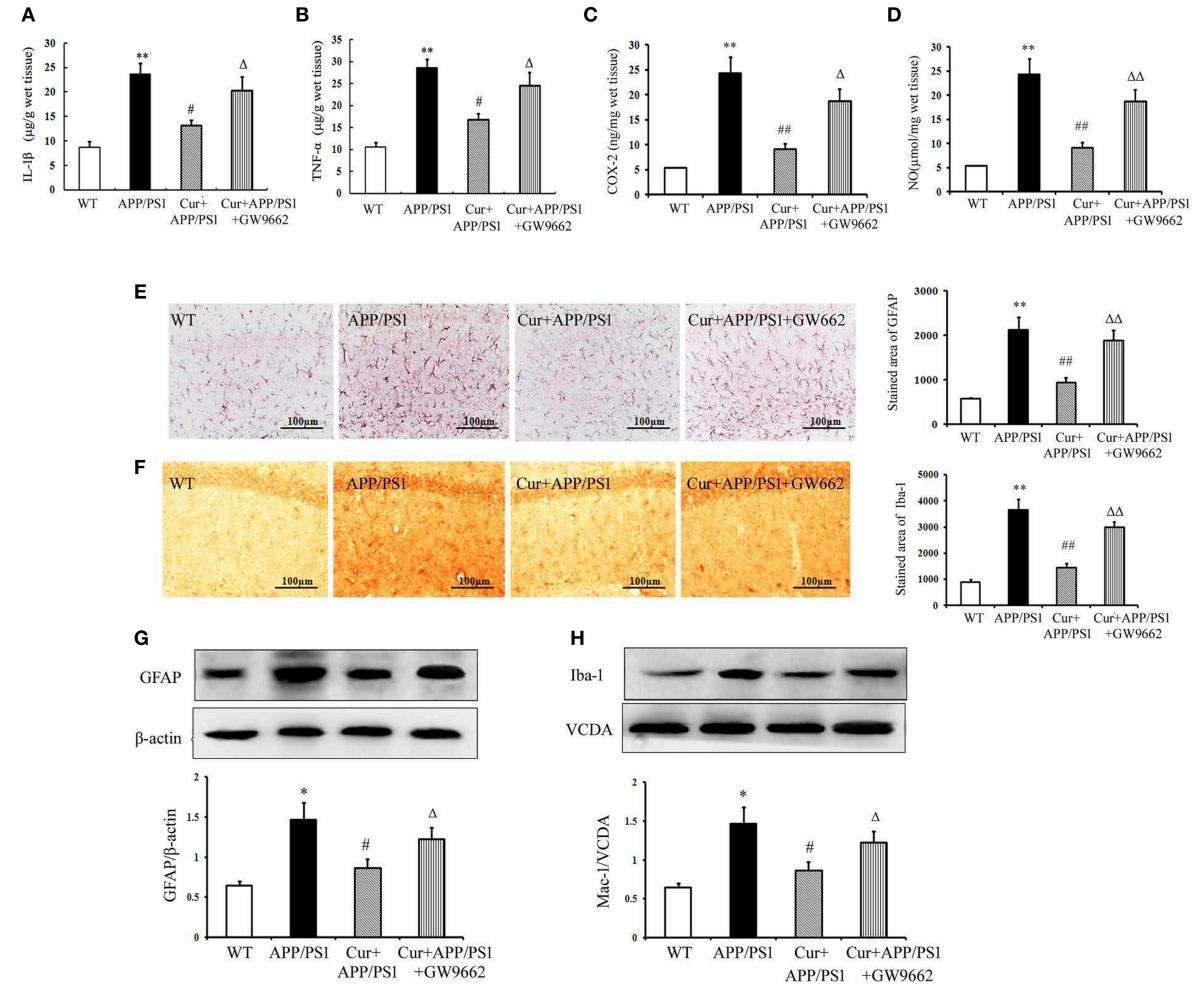
**Curcumin suppressed neuroinflammatory response in APP/PS1 transgenic mice**. Curcumin 150 mg/kg and PPARγ inhibitor GW9662 4 mg/kg were i.p. injected to APP/PS1 double-transgenic mice for 4 consecutive weeks. **(A)** IL-1β level in hippocampus. **(B)** TNF-α level in hippocampus. **(C)** COX-2 level in hippocampus. **(D)** NO level in hippocampus. Data were expressed as mean ± SD with six individual experiments. **(E)** Immunohistochemistry of GFAP in hippocampus. **(F)** Immunohistochemistry of Iba-1 in hippocampus. Representative sections of hippocampus from five mice were shown. **(G)** Western blot of GFAP in hippocampus. **(H)** Western blot of Iba-1 in hippocampus. A representative immunoblot from four mice was shown. Results were expressed as mean ± SD. ^*^P < 0.05, ^**^P < 0.01 vs. WT mice, ^#^*P* < 0.05, ^##^*P* < 0.01 vs. APP/PS1 transgenic mice, ^Δ^*P* < 0.05, ^ΔΔ^*P* < 0.01 vs. curcumin treated mice.

Aβ can activate both microglia and astrocytes, which produce multiple inflammatory mediators. Our results show that the inflammatory response in the hippocampi of APP/PS1 mice was manifested by overproduction of TNF-α, IL-1β, COX-2, and NO. Treatment of mice with curcumin markedly suppressed the production of these toxic mediators (Figures 4A–D). We then examined the possible activation of microglia and astrocytes in the hippocampi of mice. In the WT mice, a small number of Iba1-positive microglia and GFAP-positive astrocytes were distributed throughout the hippocampus. Iba1-positive microglia with enlarged cell bodies increased, and the accumulation of GFAP-positive astrocytes with enlarged cell bodies and short processes was also noted in the hippocampi of APP/PS1 mice (Figures 4E,F). Furthermore, western blot showed that the expression of GFAP and Iba-1 markedly increased in APP/PS1 mice (Figures 4G,H). These morphological and expression changes strongly suggest that both microglia and astrocytes distributed in the hippocampus were activated upon Aβ stimulation. Curcumin treatment suppressed neuroinflammation, as indicated by the reduced production of inflammatory mediators, decreased number of GFAP- and Iba-1-positive cells, and expression in the hippocampus. As expected, GW9662 attenuated the anti-neuroinflammatory effects of curcumin (Figure 4).

### Curcumin inhibited neuroinflammation in mixed neuronal/glial cultures

**Figure 5:**
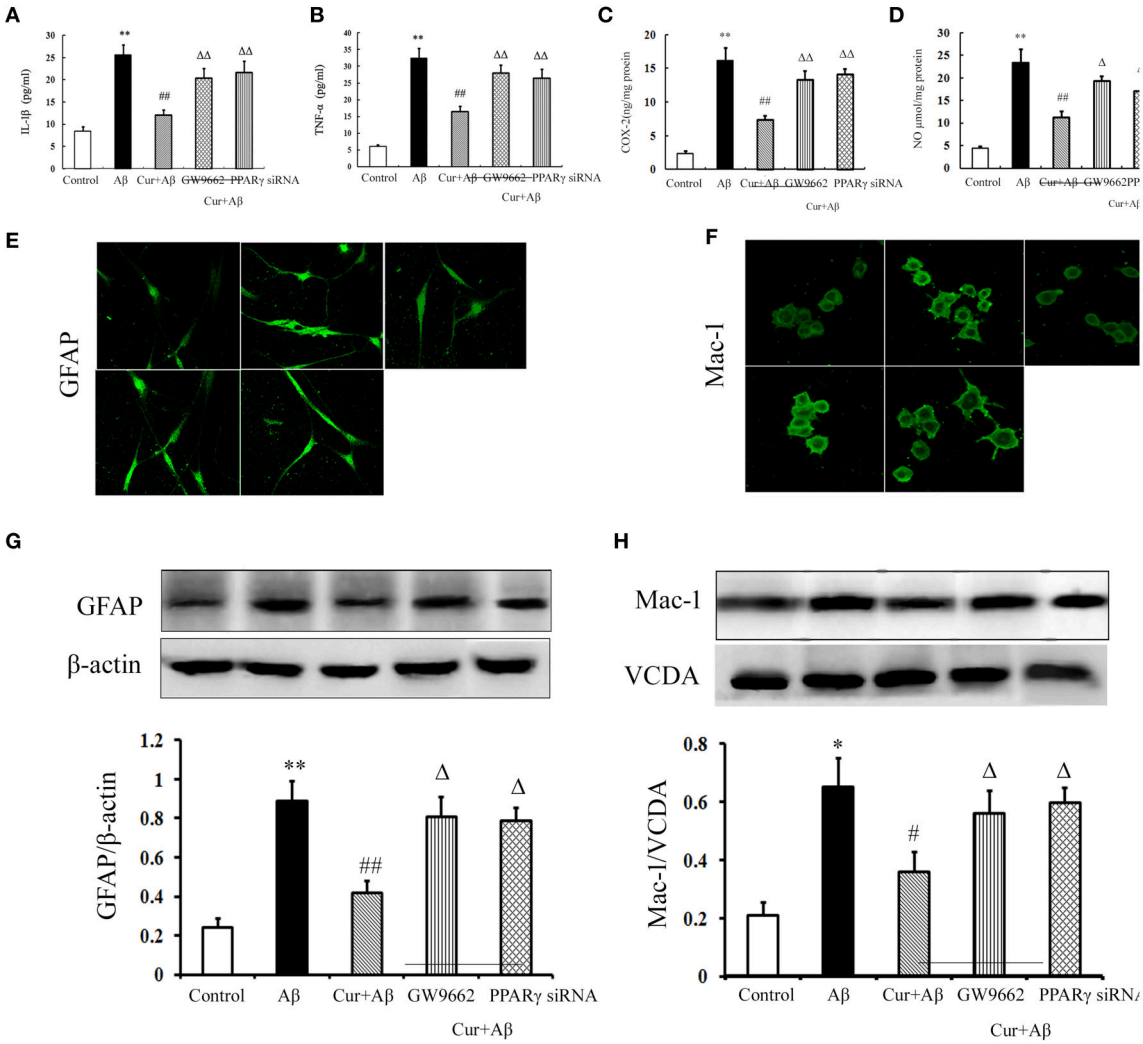
**Curcumin inhibited neuroinflammation in mixed neuron/glia cultures**. Mixed neuron/glia cultures were pre-treated with curcumin 10 μM, 1 h later, Aβ_1–42_ 25 μM was added to the mixed cultures. GW9662 1 μM was added into the cultures or cells were transfected with PPARγ siRNA 1 h before Aβ_1–42_ treatment. **(A)** IL-1β level of mixed neuron/glia cultures. **(B)** TNF-α level of mixed neuron/glia cultures. **(C)** COX-2 level of mixed neuron/glia cultures. **(D)** NO level of mixed neuron/glia cultures. Data were expressed as mean ± SD with six individual experiments. **(E)** Immunofluorescence of GFAP. **(F)** Immunofluorescence of Mac-1. Representative images from five experiments were shown. **(G)** Western blot of GFAP in mixed neuron/glia cultures. **(H)** Western blot of Iba-1 in mixed neuron/glia cultures. A representative immunoblot from four independent experiments was shown. Data were expressed as mean ± SD. ^*^P < 0.05, ^**^P < 0.01 vs. control cells, ^#^*P* < 0.05, ^##^*P* < 0.01 vs. Aβ_1–42_-challenged cells, ^Δ^*P* < 0.05, ^ΔΔ^*P* < 0.01 vs. curcumin treated cells.

We further investigated the inhibitory effects of curcumin on Aβ-induced neuroinflammation in mixed neuronal/glial cultures. Similarly, Aβ_1–42_ stimulation triggered the inflammatory response, as indicated by elevated levels of TNF-α, IL-1β, COX-2, and NO in the media. Activation of microglia and astrocytes was also observed with increased GFAP and Iba-1 immunoreactivity, as well as the expression of proteins. Curcumin treatment significantly reduced the concentrations of inflammatory mediators. Co-administration of GW9662 or silencing of PPARγ by RNAi attenuated the anti-inflammatory effects of curcumin (Figure 5). Combined with the results of the *in vivo* study, these results suggest that the treatment effects of curcumin on AD were closely related to its inhibition of neuroinflammation, which might be mediated by PPARγ.

### Curcumin suppressed the NF-κB signaling pathway

**Figure 6:**
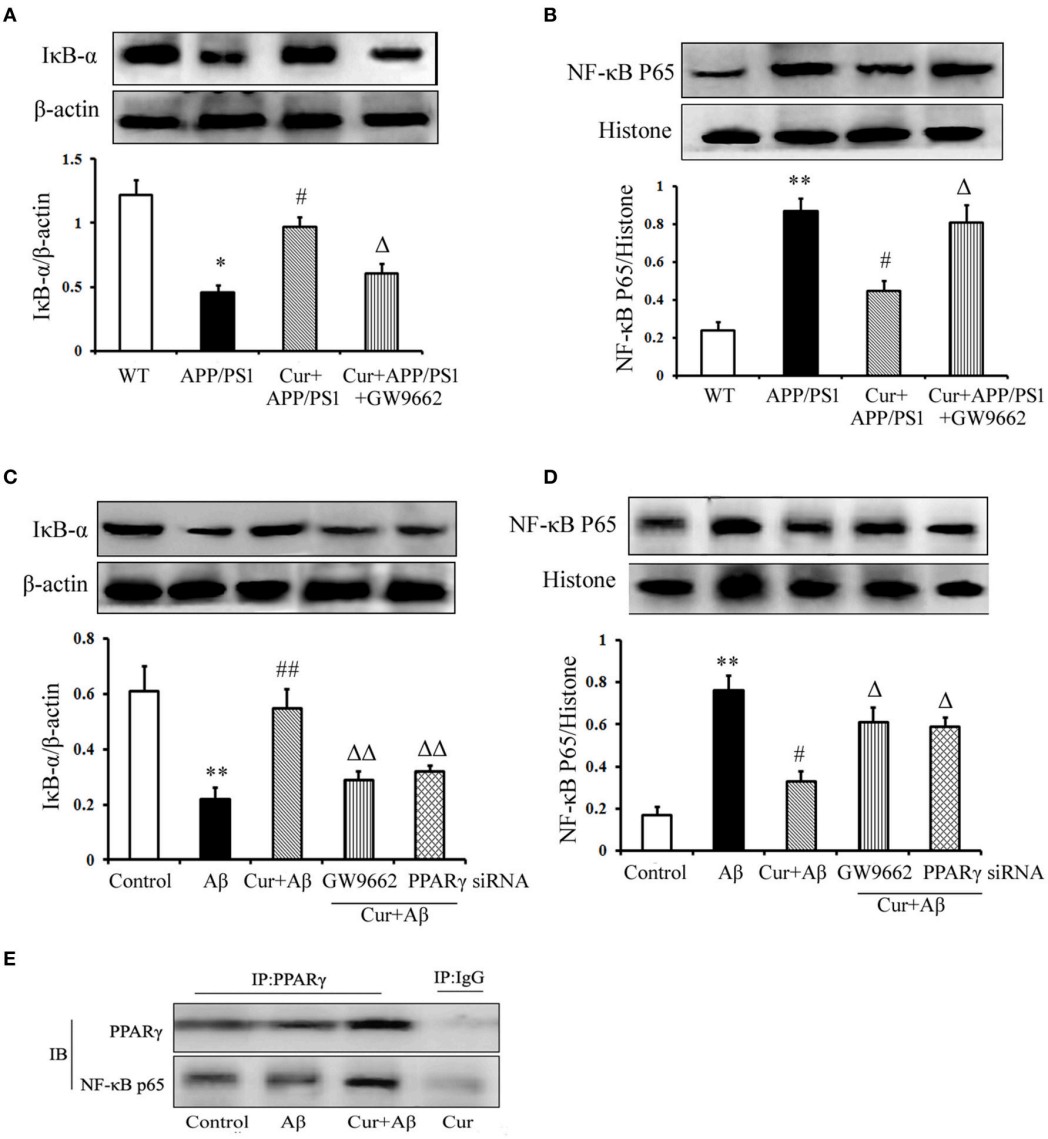
**Curcumin suppressed NF-κB signaling pathway**. Curcumin 150 mg/kg and PPARγ inhibitor GW9662 4 mg/kg were i.p. injected to APP/PS1 double-transgenic mice for 4 consecutive weeks. **(A)** IκB-α expression. **(B)** NF-κB p65 expression. Data were expressed as mean ± SD. Western blot images were representative of four mice. Results were expressed as mean ± SD. ^*^P < 0.05, ^**^P < 0.01 vs. WT mice, ^#^*P* < 0.05, vs. APP/PS1 transgenic mice, ^Δ^*P* < 0.05, vs. curcumin treated mice. Mixed neuron/glia cultures were pre-treated with curcumin 10 μM, 1 h later, Aβ_1–42_ 25 μM was added to the mixed cultures. GW9662 1 μM was added into the cultures or cells were transfected with PPARγ siRNA 1 h before Aβ_1–42_ treatment. **(C)** IκB-α expression. **(D)** NF-κB p65 expression. Data were expressed as mean ± SD. Western blot images were representative of four independent experiments. Results were expressed as mean ± SD. ^**^P < 0.01 vs. control cells, ^#^*P* < 0.05, ^##^*P* < 0.01 vs. Aβ_1–42_-challenged cells, ^Δ^*P* < 0.05, ^ΔΔ^*P* < 0.01 vs. curcumin treated cells. **(E)** Interaction of PPARγ and NF-κB p65.

Studies have shown that NF-κB signaling is involved in inflammation and the immune response, including in the brain. In APP/PS1 mice, IκB-α degradation, and NF-κB p65 translocation were observed. However, pre-treatment of curcumin decreased IκB-α degradation and NF-κB p65 translocation. The inhibitory effect of curcumin on IκB-α degradation and NF-κB p65 translocation was counteracted by co-administration of GW9662 (Figures 6A,B).

We then conducted *in vitro* experiments, in mixed neuronal/glial cultures to confirm the *in vivo* results. Our results demonstrate that increased IκB degradation and NF-κB p65 translocation were stimulated by Aβ_1–42_, which was inhibited by curcumin treatment (Figures 6C,D). Co-administration of GW9662 or transfection with 25 nM PPARγ siRNA attenuated the inhibitory effects of curcumin on NF-κB signaling.

PPARγ has been reported to inhibit NF-κB activity. Thus, we investigated the effect of PPARγ on NF-κB in mixed neuronal/glial cells stimulated with Aβ. The data show that PPARγ interacted with NF-κB p65 in curcumin-treated cells. However, blocking of PPARγ with GW9662 or PPARγ siRNA decreased the interaction of PPARγ with NF-κB p65 (Figure 6E) suggesting that PPARγ signaling was involved in the suppression of NF-κB activation in the neuroprotection of curcumin.

### Curcumin improved PPARγ function

**Figure 7:**
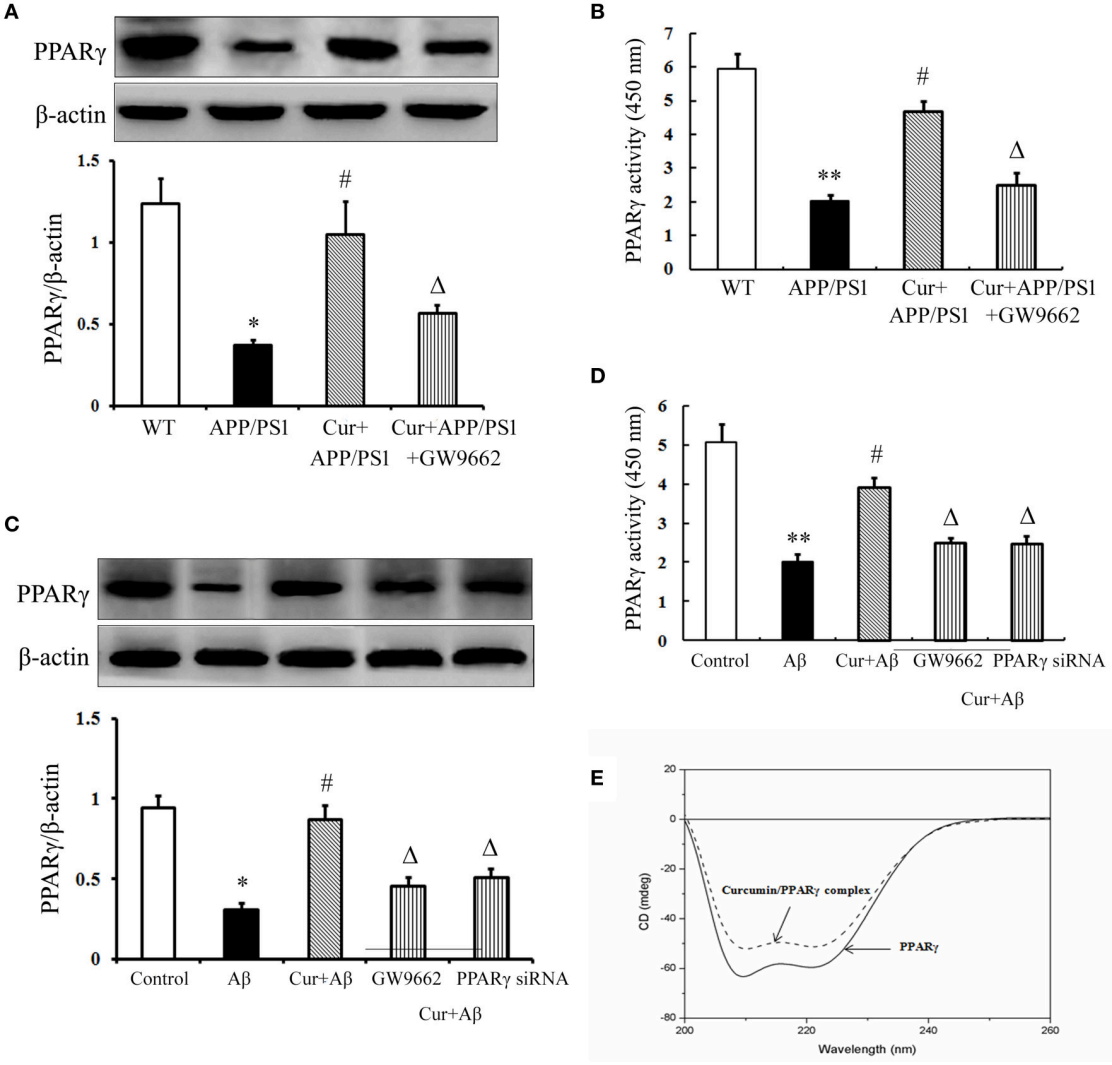
**Curcumin improved PPARγ function**. Curcumin 150 mg/kg and PPARγ inhibitor GW9662 4 mg/kg were i.p. injected to APP/PS1 double-transgenic mice for 4 consecutive weeks. **(A)** Western blot assay of PPARγ expression. The Western blot images were representative of four mice. **(B)** PPARγ transcriptional activity assay. Data were expressed as mean ± SD with six individual experiments. ^*^P < 0.05, ^**^P < 0.01 vs. WT mice, ^#^*P* < 0.05, vs. APP/PS1 transgenic mice, ^Δ^*P* < 0.05, vs. curcumin treated mice. Mixed neuron/glia cultures were pre-treated with curcumin 10 μM, 1 h later, Aβ_1–42_ 25 μM was added to the mixed cultures. GW9662 1 μM was added into the cultures or cells were transfected with PPARγ siRNA 1 h before Aβ_1–42_ treatment. **(C)** Western blot assay of PPARγ expression. The Western blot images were representative of four independent experiments. **(D)** PPARγ transcriptional activity assay. Data were expressed as mean ± SD with six individual experiments. ^*^P < 0.05, ^**^P < 0.01 vs. control cells, ^#^*P* < 0.05 vs. Aβ_1–42_-challenged cells, ^Δ^*P* < 0.05 vs. curcumin treated cells. **(E)** Circular dichroism spectra of PPARγ (0.6 μM, solid line) and curcumin/ PPARγ complex (dash line) at 37°C.

The above data demonstrated that PPARγ was involved in the anti-inflammatory effects of curcumin *in vivo* and *in vitro*. Further experiments were conducted to investigate how PPARγ participated in the anti-inflammatory process. PPARγ expression and activity were obviously decreased in the hippocampi of APP/PS1 mice. The same results were obtained in primary mixed neuronal/glial cultures, suggesting that Aβ aggregation deteriorated PPARγ function. Curcumin produced a two-fold increase in PPARγ transcriptional activity, together with a significant induction of PPARγ protein expression both *in vivo* and *in vitro* (Figures 7A–D). These results suggest that curcumin was a potent agent to promote PPARγ activity. Using CD spectra technology, we further examined whether curcumin can directly bind to PPARγ. The curve showed that 1 μM curcumin could directly bind PPARγ (Figure 7E), which may explain why curcumin could improve PPARγ function. However, how curcumin bind PPARγ need to be further investigation.

## Discussion

In this study, we performed a series of *in vivo* and *in vitro* experiments demonstrating that curcumin could alleviate spatial memory deficits and promote cholinergic neuronal function. The beneficial effects of curcumin on AD were due to the suppression of neuroinflammation, as indicated by the reduced activation of glia and cytokine production, as well as inhibition of the NF-κB signaling pathway. In addition, this compound produced a two-fold increase in PPARγ transcriptional activity, together with a significant induction of PPARγ protein expression. Notably, curcumin directly bound to PPARγ and upregulated its function. These data together suggest that the modulation of PPARγ activity by curcumin may contribute to alleviated neuroinflammation and improved neuronal function.

Neuroinflammation associated with AD is often viewed as a secondary response to Aβ deposition and neuronal death, but plays a pivotal role in the pathogenesis and development of AD (Amor et al., 2010). Microglia and astrocytes are activated in response to Aβ, and they communicate with each other in a bidirectional manner. Activated glia in senile plaques can secrete vast amounts of pro-inflammatory mediators, such as cytokines and chemokines, which are toxic to neurons (Agostinho et al., 2010). It was reported that there were high levels of IL-1β and TNF-α in brain and cerebrospinal fluid of AD patients (Angelopoulos et al., 2008; Forlenza et al., 2009), which provided evidence the role of inflammation in the etiology of AD. In the animal model of AD, microglia and astrocytes mediated neuroinflammation contribute the production and formation of Aβ aggregates (Morales et al., 2010). Thus, AD may possibly be treated by modulating glial function and suppressing the inflammatory response in the brain. A pharmacokinetics study showed that curcumin can cross the blood-brain barrier, where it is concentrated chiefly in the hippocampus (Tsai et al., 2011). Moreover, curcumin is a potent reagent for the treatment of AD (Wang et al., 2013). In the present study, we demonstrated the robust activation of astrogliosis and microgliosis, as well as a strong increase in IL-1β, TNF-α, COX-2, and NO in the hippocampi of APP/PS1 transgenic mice and mixed neuronal/glial cultures. These findings confirm that the inflammatory response is involved in the pathogenesis of AD. As expected, administration of curcumin suppressed reactive gliosis as indicated by reducing cytokine release. Given that neuroinflammation is important in the development of neurodegenerative disease, the *in vivo* and *in vitro* anti-inflammation of curcumin may provide additional evidence of its therapeutic potential in AD.

The important role of PPARγ agonists in neuroprotection has been extensively studied in neurodegeneration, such as in Aβ-induced AD (Bright et al., 2008). Activation of PPARγ signaling was shown to enhance Aβ uptake by microglia and thus improve cognitive function in AD mice (Yamanaka et al., 2012). In our previous study, we demonstrated that curcumin was a potent agent for promoting PPARγ activity, which played a critical role in protecting against cerebral ischemic injury because of its ability to suppress the inflammatory response (Liu et al., 2013, 2014). In the present study, curcumin elicited a two-fold increase in the transcriptional activity of PPARγ and prompt expression of PPARγ protein, thereby indicating the up-regulated activity of PPARγ. Curcumin could directly bind to PPARγ protein, which might be the basis of its anti-inflammatory activity. Moreover, the reduction of NO, TNF-α, and IL-1β production by curcumin was accompanied by the marked decline of GFAP and Iba-1 expression, which may correlate to the activation of PPARγ. The observation that activation of PPARγ contributed to the decrease in IκB degradation and NF-κB p65 protein translocation further reinforced the importance of PPARγ on the inhibition of NF-κB as responsible for curcumin's anti-inflammatory effects. Notably, PPARγ was involved in neuroprotective and anti-gliotic effects both *in vivo* and *in vitro*, because a loss in beneficial activity occurred upon co-administration with PPARγ antagonist GW9662 or blocking the expression of PPARγ by RNAi. Our study confirmed previous reports that overexpression of PPARγ can protect neurons from injury (Mandrekar-Colucci et al., 2012; Jahrling et al., 2014). Therefore, PPARγ might be an important mechanism responsible for the anti-inflammatory effects of curcumin.

NF-κB plays a vital role in regulating of inflammation in many diseases including brain injury and neurodegenerative diseases (Song et al., 2004; Samuelsson et al., 2005). Study has shown that curcumin could inhibit the activity of NF-κB through reducing IκB-α degradation (Moon et al., 2006). Similarly, in the present study, we observed significantly increased IκB-α degradation and NF-κB p65 translocation in APP/PS1 mice and mixed neuronal/glial cultures stimulated by Aβ, whereas curcumin effectively reversed the activated NF-κB signaling. These results suggest that the suppression of Aβ-triggered inflammation by curcumin was through inhibition of the NF-κB signaling pathway, which consistent with others studies demonstrating, the inhibitory effects of PPARγ on NF-κB activation in different cell systems. Activation of NF-κB is critically regulated at multiple steps. In the current study, PPARγ physically interacted with the NF-κB p65 subunit, blocked NF-κB activation, and inhibited the dependent gene expression. Notably, PPARγ activation and upregulation by curcumin were crucial to its inhibitory action on NF-κB because the effects abated in part with co-administration of GW9662 or silence of PPARγ. The present results confirmed the results of our previous study, which showed that NF-κB activity was inhibited by PPARγ in *in vivo* and *in vitro* cerebral ischemic models. Moreover, the present data were supported by the finding that PPARγ has been detected in the hippocampi of adult rats (Moreno et al., 2004), and PPARγ activation is reported to suppress inflammatory gene expression because of the inhibition of NF-κB in animal models of brain damage (Collino et al., 2006). Therefore, we speculated that activation of PPARγ by curcumin may be a key step in inhibition of NF-κB signaling pathway.

In summary, the curcumin data verified previous reports demonstrating that neuroinflammation is risk factor in the development of AD, and curcumin showed beneficial effects on AD through suppressing such inflammatory response. The present study demonstrated that the improvement of curcumin on memory deficits in AD might be through activation of PPARγ pathway, which mitigates the neuroinflammatory response via inhibiting the NF-κB signaling pathway.

## Author contributions

CX and Z-JL formulated the concept and designed the manuscript. Z-JL, LL, WT, and YW performed the experiments. Z-JL, Z-HL, and LL analyzed the data. Z-JL and YW drafted the manuscript. LL, WT, YW, MD, and CX participated in discussions related to the paper. Z-HL, CX, WT, and YW revised the manuscript. All of the authors read and approved the final manuscript.

### Conflict of interest statement

The authors declare that the research was conducted in the absence of any commercial or financial relationships that could be construed as a potential conflict of interest.

## Supplementary material

Figure S1 **Morris water maze test in 8-month-old APP/PS1 transgenic mice**. ^**^*P* < 0.01 vs. WT mice.

Figure S2 **Aβ accumulation in the hippocampi of 8-month-old APP/PS1 transgenic mice**.

Figure S3 **GW9662 (4 mg/kg) did not influence memory of APP/PS1 mice**. ^*^*P* < 0.05 vs. WT mice.

Figure S4 **GW9662 (4 mg/kg) did not influence neuronal function of APP/PS1 mice**. ^*^*P* < 0.05 vs. WT mice.

Figure S5 **GW9662 or PPARγ siRNA alone did not affect cholinergic neuronal function**.
