# Supplementary figures and images for: Curcumin Attenuates Beta-Amyloid-Induced Neuroinflammation via Activation of Peroxisome Proliferator-Activated Receptor-Gamma Function in a Rat Model of Alzheimer's Disease
Source: Front Pharmacol. 2016 Aug 19;7:261. doi: 10.3389/fphar.2016.00261 (PMC4990744; doi:10.3389/fphar.2016.00261)

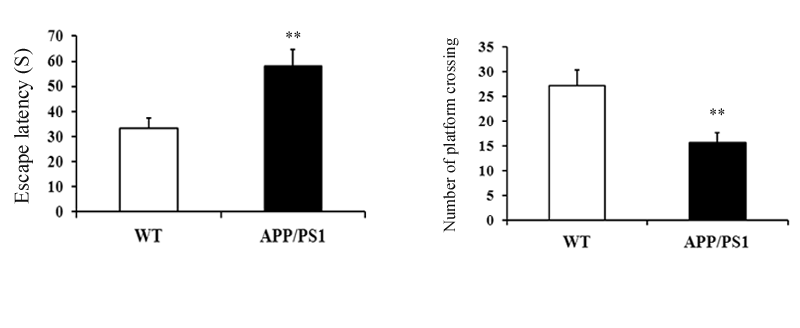

Supplement: Figure S1 — Morris water maze test in 8-month-old APP/PS1 transgenic mice. **P < 0.01 vs. WT mice. [file Image1.TIF]

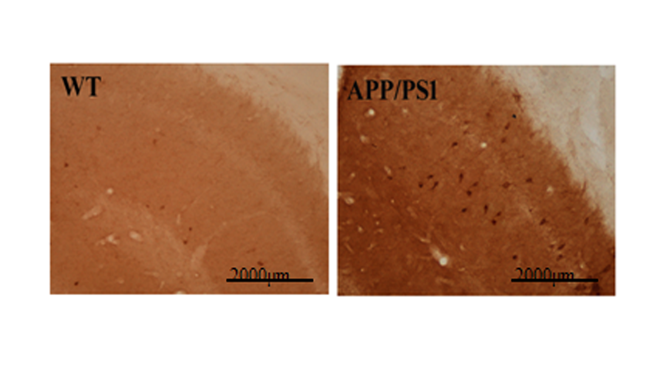

Supplement: Figure S2 — Aβ accumulation in the hippocampi of 8-month-old APP/PS1 transgenic mice. [file Image2.TIF]

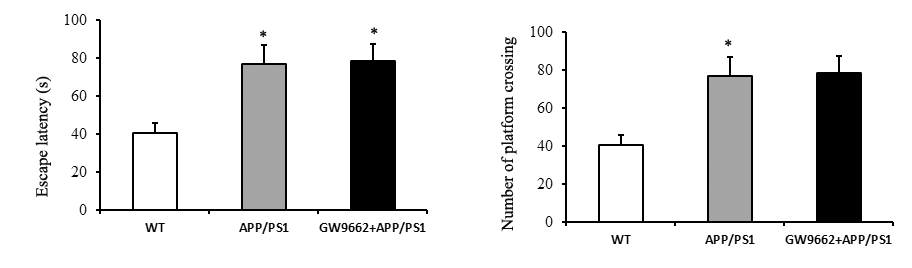

Supplement: Figure S3 — GW9662 (4 mg/kg) did not influence memory of APP/PS1 mice. *P < 0.05 vs. WT mice. [file Image3.tif]

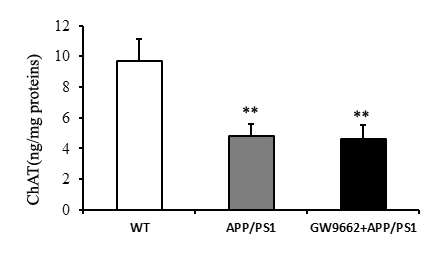

Supplement: Figure S4 — GW9662 (4 mg/kg) did not influence neuronal function of APP/PS1 mice. *P < 0.05 vs. WT mice. [file Image4.TIF]

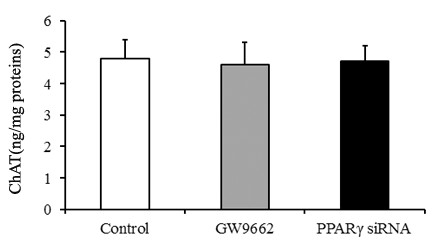

Supplement: Figure S5 — GW9662 or PPARγ siRNA alone did not affect cholinergic neuronal function. [file Image5.TIF]
